# A Challenging Case of Acute Methotrexate Toxicity

**DOI:** 10.1002/ccr3.73437

**Published:** 2026-08-31

**Authors:** Sahel Asgari, Narges Alizadeh, Shirin Zaresharifi, Mohaya Farzin

**Affiliations:** ^1^ Department of Internal Medicine, School of Medicine Poursina Hospital, Guilan University of Medical Sciences Rasht Iran; ^2^ Department of Dermatology, Skin Research Center Razi Hospital, School of Medicine, Guilan University of Medical Sciences Rasht Iran; ^3^ Department of Dermatology, Skin Research Center Shahid Beheshti University of Medical Sciences Tehran Iran; ^4^ Razi Clinical Research Development Unit Razi Hospital, Guilan University of Medical Sciences Rasht Iran

**Keywords:** drug eruption, methotrexate toxicity, neutropenic enter colitis, pancytopenia, psoriasis

## Abstract

Catastrophic methotrexate toxicity, characterized by life‐threatening pancytopenia and neutropenic enterocolitis, may occur even after a single low therapeutic dose, particularly in patients with renal impairment or dehydration. Early recognition and prompt multidisciplinary management are essential to improve outcomes.

## 1 Introduction

Methotrexate (MTX) remains a cornerstone in the management of autoimmune and proliferative disorders such as psoriasis, rheumatoid arthritis, and certain malignancies, owing to its potent anti‐proliferative and anti‐inflammatory properties [1, 2, 3]. When used in low weekly doses, MTX is generally well tolerated and regarded as safe; however, serious systemic toxicity can occur even within the therapeutic range, often unpredictably and with potentially fatal outcomes [4, 5, 6].

MTX acts as an inhibitor of dihydrofolate reductase, impairing tetrahydrofolate formation and thereby halting DNA synthesis in rapidly dividing cells. As a result, tissues with high turnover—such as bone marrow, mucosal surfaces, and epidermis—are most vulnerable to its cytotoxic effects [2, 3]. The pharmacokinetics of MTX depend heavily on renal clearance and folate metabolism; thus, even mild renal impairment, dehydration, or folate deficiency can drastically increase systemic exposure [3, 4, 7].

Although the standard weekly low‐dose regimen has proven efficacy in dermatologic and rheumatologic conditions, toxic events such as pancytopenia, mucocutaneous ulceration, and hepatic or gastrointestinal injury have been increasingly reported in recent literature [1, 5, 6, 8]. According to several recent case reports and reviews, the onset of toxicity may be abrupt and severe, sometimes appearing after only a few doses—or, rarely, after the first administration—with mortality rates up to 20%–30% in untreated cases [3, 4, 6].

Furthermore, dermatologic manifestations such as painful erosions, bullous eruptions, or TEN‐like lesions are being recognized as early indicators of systemic MTX toxicity, often preceding hematologic collapse [7, 8]. This highlights the importance of early clinical suspicion and laboratory monitoring, especially in patients with impaired renal function, advanced age, or potential dosing errors [3, 6, 9].

Herein, we report a severe case of acute methotrexate toxicity following a single intramuscular dose prescribed for suspected psoriasis, presenting with widespread mucocutaneous eruptions, pancytopenia, and probable neutropenic enterocolitis. This case underscores the unpredictable nature of MTX toxicity, the necessity for prompt recognition, and the crucial role of early multidisciplinary intervention.

## 2 Case Presentation

The patient, a 32‐year‐old man, presented with fine scaly plaques on the extensor surface of the left elbow that had appeared approximately 20 days before admission. With a clinical suspicion of psoriasis, he was started on methotrexate therapy. Following the first intramuscular dose of methotrexate (10 mg), the patient developed numerous pustular and bullous cutaneous and mucosal lesions distributed across the entire body and mucosal surfaces, along with facial and lip swelling, oral mucosal erosions and crust formation, conjunctival congestion and redness, generalized weakness, malaise, fever, and recurrent nausea and vomiting. He presented to the emergency department in poor general condition.

Upon admission, the patient appeared severely ill, dehydrated, and toxic. His blood pressure was low (BP: 80/60 mmHg), with filiform and tachycardic pulses (PR: 140 bpm) and a fever of 39°C (Figures 1 and 2).

**FIGURE 1:**
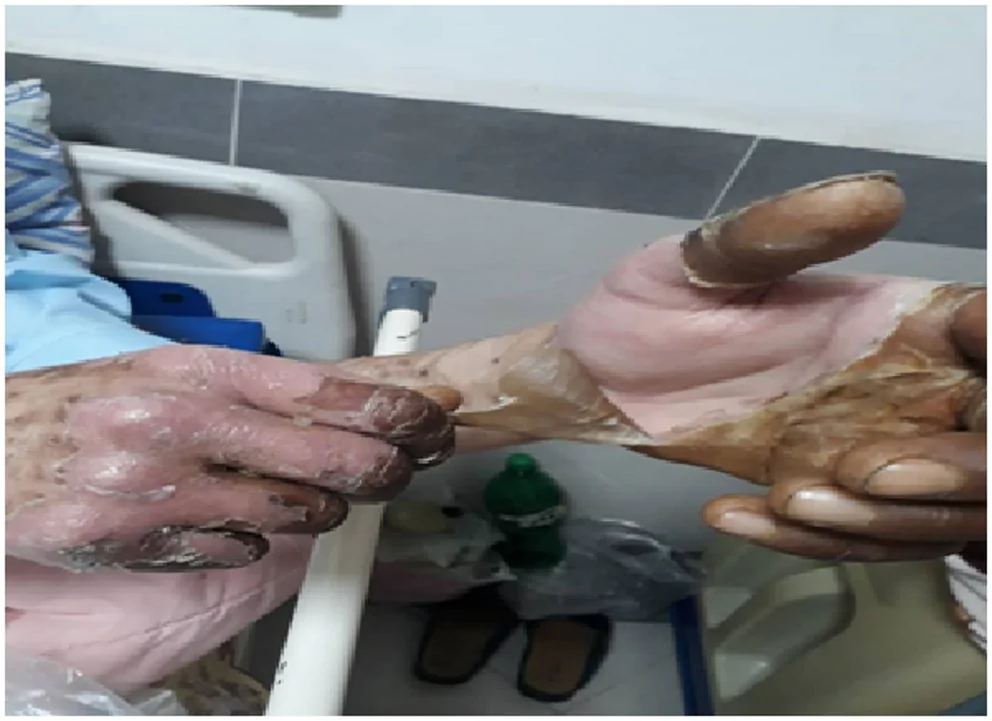
Severe epidermal necrolysis and desquamation on the patient's hands following single‐dose methotrexate toxicity. The peeling of epidermal layers and dark discoloration are indicative of advanced mucocutaneous involvement.

**FIGURE 2:**
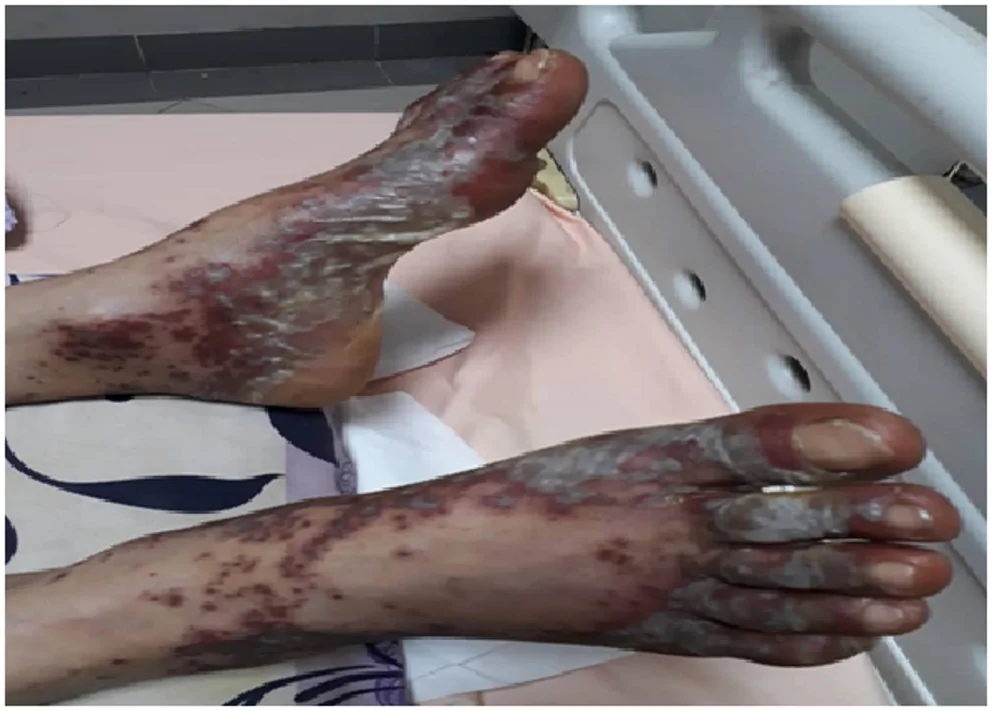
Widespread ulcerative and necrotic lesions on both feet and lower legs, associated with methotrexate‐induced cutaneous toxicity. The distribution and morphology resemble toxic epidermal necrolysis.

A review of the patient's medical history revealed no prior illnesses or medication use, except for taking methadone tablets at a daily dose of 5 mg.

### 2.1 Laboratory Findings

The patient's laboratory results are summarized below.

### 2.2 Biochemistry Results

The biochemical tests showed severe metabolic acidosis (pH: 7.19, HCO_3_: 18.1), elevated liver enzymes (ALT: 350 IU/L, AST: 460 IU/L, ALP: 160 IU/L, CPK: 1030 IU/L, LDH: 9080 IU/L), markedly increased inflammatory markers (ESR: 88 mm/h, CRP: 299 mg/L), and evidence of renal dysfunction (BUN: 38 mg/dL, Creatinine: 1.72 mg/dL) (Table 1).

**TABLE 1:** Biochemistry results.

Parameter	Result	Reference range
pH	7.19	7.35–7.45
PaCO_2_ (mmHg)	51.7	35–45
HCO_3_^−^ (mmol/L)	18.1	22–26
Blood sugar (mg/dL)	126	70–100
Blood urea nitrogen (BUN) (mg/dL)	38	7–21
Creatinine (mg/dL)	1.72	0.6–1.2
Sodium (Na) (mmol/L)	137	135–145
Potassium (K) (mmol/L)	4.0	3.5–4.5
Calcium (mg/dL)	8.3	8.6–10.3
Magnesium (mg/dL)	1.7	1.8–2.6
Lactate dehydrogenase (LDH) (U/L)	9080	240–480
Alanine aminotransferase (ALT) (U/L)	350	< 40
Aspartate aminotransferase (AST) (U/L)	460	< 40
Alkaline phosphatase (ALP) (U/L)	160	120–290
Creatine phosphokinase (CPK) (U/L)	1030	< 174
Albumin (g/dL)	3.3	3.5–5.2
Erythrocyte sedimentation rate (ESR) (mm/h)	88	< 30
C‐reactive protein (CRP) (mg/L)	299	< 10
Total bilirubin (mg/dL)	2.0	0.3–1.0
Direct bilirubin (mg/dL)	0.2	0.1–0.3
Urine RBC (HPF)	2–3	0–2
Urine WBC (HPF)	3–4	0–5

### 2.3 Hematology Results

Hematologic evaluation revealed leukopenia (WBC: 0.53 × 10^9^/L), anemia (Hb: 9.7 g/dL), and thrombocytopenia (PLT: 102 × 10^9^/L), consistent with early pancytopenia (Table 2).

**TABLE 2:** Hematology results.

Parameter	Result	Reference range
White blood cell count (×10^9^/L)	0.53	4.0–11.0
Red blood cell count (×10^12^/L)	3.31	4.5–5.9
Hemoglobin (g/dL)	9.7	13.0–17.5
Hematocrit (%)	29.6	42–52
Mean corpuscular volume (MCV) (fL)	89.4	80–100
Mean corpuscular hemoglobin (MCH) (pg)	29.3	27–33
Mean corpuscular hemoglobin concentration (MCHC) (g/dL)	32.8	31–37
Platelet count (×10^9^/L)	102	150–450

Treatment was initiated immediately. The patient received intravenous fluid therapy (4–6 L per 24 h), leucovorin injections, antihistamines, hydrocortisone, and antibiotics. He was transferred to an isolation room and placed under continuous monitoring.

On the second day of hospitalization, the patient's cutaneous lesions continued to spread. Despite all therapeutic measures, his general condition worsened, with recurrent vomiting, severe anorexia, severe oral ulcerations, odynophagia, dysphagia, and progression of pancytopenia. His white blood cell count dropped to 100/μL, hemoglobin to 6 g/dL, and platelets to 40,000/μL.

A peripheral blood smear was prepared and reviewed by a hematologist, who confirmed the absence of abnormal cells. Treatment with G‐CSF was initiated, and further serological testing was performed to investigate the causes of pancytopenia. The dermatologist performed a skin biopsy of the lesions, and smear and culture samples were obtained from the skin discharge.

On the third day of hospitalization, the patient remained critically ill and complained of severe generalized abdominal pain. On examination, there was diffuse abdominal tenderness and distention. Surgical consultation and complete abdominal and pelvic ultrasonography were performed, raising suspicion of neutropenic enterocolitis. The patient was evaluated daily by an internal medicine team, along with nephrology, hematology, dermatology, and infectious disease specialists.

By the fifth day of hospitalization, despite receiving two doses of G‐CSF, the white blood cell count remained below 100/μL. Supportive treatments—including full‐dose leucovorin, hydration, local oral and mucosal care, broad‐spectrum antibiotics, proton‐pump inhibitors, and other supportive measures—were continued. Although his general condition began to improve after 1 week, severe pancytopenia persisted.

Due to lack of hematologic recovery, and following hematology consultation as well as negative serologic tests for infectious and rheumatologic causes, bone marrow aspiration and biopsy (BMA and BMB) were performed after approximately 10 days of hospitalization.

### 2.4 Viral and Immunologic Tests

Serologic results were negative for HIV, HBsAg, and HCV. Autoimmune screening showed normal complement levels (C3: 119 mg/dL, C4: 33 mg/dL) and negative ANA (Table 3).

**TABLE 3:** Viral markers and immunologic tests.

Parameter	Result	Reference range
HIV antibody	Non‐reactive	< 0.9 (Non‐reactive)
Hepatitis B surface antigen (HBsAg)	Non‐reactive	< 0.9 (Non‐reactive)
Hepatitis C antibody (HCV Ab)	Non‐reactive	< 0.9 (Non‐reactive)
Antinuclear antibody (ANA)	0.2 (Negative)	< 0.9 (Negative)
Complement C3 (mg/dL)	119	75–135
Complement C4 (mg/dL)	33	9–36

### 2.5 Biopsy

The bone marrow biopsy revealed a trilineage population of hematopoietic cells with normal maturation. No non‐native elements were observed, and a slight increase in plasmacytoid cells was noted.

The skin biopsy report was consistent with a *drug eruption*. Blood, urine, and skin culture results were all negative.

From the beginning of the third week of hospitalization, the patient's lesions began to improve, fever subsided, and laboratory results showed a gradual trend toward recovery. The CBC differential progressively normalized, with increases in white blood cell, platelet, and hemoglobin levels. The patient's appetite also improved, and after 20 days, he was discharged in good general condition with WBC = 14,000/μL, Hb = 10 g/dL, and PLT = 140,000/μL.

During subsequent follow‐up visits, including repeated CBC differential testing and clinical evaluations at the internal medicine outpatient clinic, the patient showed complete resolution of the lesions and normalization of laboratory findings.

## 3 Discussion

This case demonstrates severe, multisystem toxicity after a single low‐dose intramuscular methotrexate (MTX) injection given for presumed psoriasis. The presentation included extensive pustular–bullous mucocutaneous lesions, profound pancytopenia, and probable neutropenic enterocolitis.

Although neutropenic enterocolitis was considered the most likely diagnosis because of the close temporal relationship with methotrexate administration, profound pancytopenia, and the clinical course, inflammatory bowel disease (IBD), which may coexist with psoriasis, should also be considered in the differential diagnosis. In our patient, there was no previous history suggestive of IBD, and the overall presentation strongly favored methotrexate‐induced toxicity. Fecal calprotectin could have been a useful adjunctive investigation to distinguish between these entities and should be considered in similar future cases.

Although low‐dose MTX is widely used for dermatologic and rheumatologic diseases, numerous recent reports highlight that even small doses can produce life‐threatening complications when risk factors coexist [1, 4, 5].

### 3.1 Mechanism and Risk Factors

MTX inhibits dihydrofolate reductase, suppressing DNA synthesis in rapidly dividing tissues such as bone marrow and mucosa. Impaired renal clearance, dehydration, folate deficiency, and drug interactions are key drivers of toxicity [2, 6]. Our patient's elevated creatinine (1.72 mg/dL) and dehydration likely reduced drug elimination, leading to accumulation and marrow failure. Several 2024–2025 reviews confirm that acute kidney injury and folate deficiency remain the strongest independent predictors of pancytopenia [3, 4].

### 3.2 Clinical Pattern and Dermatologic Involvement

The patient's diffuse pustular and bullous eruptions with mucositis correspond to cutaneous signs of systemic MTX toxicity. Recent case reports describe TEN‐like epidermal necrolysis and drug‐induced pustular eruptions caused by MTX, histologically labeled as “drug eruption,” as in our case [8, 10]. These lesions may mimic severe cutaneous adverse reactions (SCARs) such as Stevens–Johnson syndrome or AGEP, and biopsy remains essential for differentiation [7, 8].

### 3.3 Pancytopenia and Hematologic Course

Profound pancytopenia developed within days of the first dose (WBC ≈100/μL, Hb ≈6 g/dL, PLT ≈40,000/μL). This mirrors recent reports showing that marrow suppression can appear after only one or two doses of MTX [1, 4, 9]. Early leucovorin rescue, G‐CSF, and supportive therapy remain the cornerstone of management [2, 3]. Recent series emphasize that unintentional daily dosing instead of weekly remains a leading cause of fatal pancytopenia [3, 6].

### 3.4 Neutropenic Enterocolitis

Abdominal pain and distension in this neutropenic patient raised suspicion for neutropenic enterocolitis. A 2024 report described MTX‐induced life‐threatening neutropenic enterocolitis successfully treated with IVIG and aggressive supportive care [4]. Although evidence is limited, consensus recommendations include bowel rest, broad‐spectrum antibiotics, and surgical evaluation for complications [2, 4].

### 3.5 Preventive Strategies

Across recent reviews, preventable causes of toxicity include dosing errors, delayed laboratory monitoring, and poor patient education [1, 3, 6]. The literature recommends: Mandatory weekly‐only labeling of MTX. Baseline and early follow‐up of CBC, creatinine, and liver enzymes. Patient counseling about early warning signs (mucositis, rash, fatigue). Standardized institutional protocols for leucovorin rescue and G‐CSF initiation [2, 3].

### 3.6 Clinical Implication

This case underscores that catastrophic MTX toxicity may occur even after a *single low dose* in a young, otherwise healthy adult. The patient's full recovery following prompt intervention supports current evidence that early recognition and multidisciplinary management substantially improve outcomes [3, 5, 9].

## 4 Conclusion

Methotrexate (MTX), though considered safe in low weekly doses, can lead to sudden and life‐threatening systemic toxicity even after a single therapeutic dose. This case highlights the importance of recognizing early mucocutaneous and hematologic warning signs, as prompt identification and initiation of leucovorin rescue, G‐CSF support, hydration, and infection control are critical to survival.

Recent literature confirms that renal dysfunction, dehydration, folate deficiency, and medication errors—particularly daily instead of weekly dosing—remain the predominant risk factors. Clinicians should emphasize strict patient education, routine monitoring of renal and hematologic parameters, and interdisciplinary coordination between dermatology, internal medicine, nephrology, and hematology teams.

Ultimately, early recognition and aggressive supportive management can lead to full recovery, as demonstrated in this patient, reinforcing that vigilance is essential whenever methotrexate is prescribed, regardless of dose or indication.

## Author Contributions

**Sahel Asgari:** conceptualization, supervision, writing – review and editing. **Narges Alizadeh:** data curation, writing – original draft. **Shirin Zaresharifi:** data curation, writing – original draft. **Mohaya Farzin:** data curation, funding acquisition, writing – original draft, writing – review and editing.

## Funding

The authors have nothing to report.

## Ethics Statement

This study was approved by the Ethics Committee of Guilan University of Medical Sciences (Approval No. IR.GUMS.REC.1404.214).

## Consent

Written informed consent was obtained from the patient for publication of this case report and accompanying images.

## Conflicts of Interest

The authors declare no conflicts of interest.

## Data Availability

The data that support the findings of this study are available on request from the corresponding author. The data are not publicly available due to privacy or ethical restrictions.
